# Abnormal left atrial body stiffness is predicted by appendage size: impact of appendage occlusion on left atrial mechanics assessed by pressure-volume analysis

**DOI:** 10.1152/ajpheart.00083.2022

**Published:** 2022-08-12

**Authors:** Alda Bregasi, James V. Freeman, Jeptha P. Curtis, Joseph G. Akar, Xochitl A. Ortiz-Leon, Julia H. Maia, Angela Y. Higgins, Ray V. Matthews, Albert J. Sinusas, Robert L. McNamara, Lissa Sugeng, Ben A. Lin

**Affiliations:** ^1^Division of Cardiovascular Medicine, Department of Internal Medicine, Keck School of Medicine of University of Southern California, Los Angeles, California; ^2^Section of Cardiovascular Medicine, Department of Internal Medicine, Yale University School of Medicine, New Haven, Connecticut; ^3^National Institute of Cardiology Ignacio Chavez, Mexico City, Mexico; ^4^Department of Radiology and Biomedical Imaging, Yale University School of Medicine, New Haven, Connecticut; ^5^Department of Biomedical Engineering, Yale University School of Engineering and Applied Science, New Haven, Connecticut

**Keywords:** atrial appendage, atrial fibrillation, atrial function, compliance, stiffness

## Abstract

Atrial cardiomyopathy has been recognized as having important consequences for cardiac performance and clinical outcomes. The pathophysiological role of the left atrial (LA) appendage and the effect of percutaneous left atrial appendage occlusion (LAAO) upon LA mechanics is incompletely understood. We evaluated if changes in LA stiffness due to endocardial LAAO can be detected by LA pressure-volume (PV) analysis and whether stiffness parameters are associated with baseline characteristics. Patients undergoing percutaneous endocardial LAAO (*n* = 25) were studied using a novel PV analysis using near-simultaneous three-dimensional LA volume measurements by transesophageal echocardiography (TEE) and direct invasive LA pressure measurements. LA stiffness (dP/dV, change in pressure with change in volume) was calculated before and after LAAO. Overall LA stiffness significantly increased after LAAO compared with baseline (median, 0.41–0.64 mmHg/mL; *P* ≪ 0.001). LA body stiffness after LAAO correlated with baseline LA appendage size by indexed maximum depth (Spearman’s rank correlation coefficient *R*_s_ = 0.61; *P* < 0.01). LA stiffness change showed an even stronger correlation with baseline LA appendage size by indexed maximum depth (*R*_s_ = 0.70; *P* < 0.001). We found that overall LA stiffness increases after endocardial LAAO. Baseline LA appendage size correlates with the magnitude of increase and LA body stiffness. These findings document alteration of LA mechanics after endocardial LAAO and suggest that the LA appendage modulates overall LA compliance.

**NEW & NOTEWORTHY** Our study documents a correlation of LA appendage remodeling with the degree of chronically abnormal LA body stiffness. In addition, we found that LA appendage size was the baseline parameter that best correlated with the magnitude of a further increase in overall LA stiffness after appendage occlusion. These findings offer insights about the LA appendage and LA mechanics that are relevant to patients at risk for adverse atrial remodeling, especially candidates for LA appendage occlusion.

## INTRODUCTION

The left atrium (LA) plays an important role both as a modulator and marker of overall cardiac performance. Atrial cardiomyopathy has been recognized as having important clinical consequences (1). More specifically, abnormalities in LA size and function have been associated with adverse outcomes involving disease states such as congestive heart failure (CHF) and atrial fibrillation (AF) (2, 3).

LA function has separate components referred to as reservoir function, conduit function, and booster pump function. Reservoir function depends upon several interacting processes such as LA contraction and relaxation, as well as left ventricular base systolic descent. LA reservoir function is assessed by measuring LA compliance (or more commonly its inverse, LA stiffness) using the LA pressure-volume (PV) relationship. Overall, LA compliance is believed to allow the maintenance of hemodynamic equilibrium in response to perturbations (4–8). Studies have shown that the LA appendage (LAA) is more compliant than the LA body (9, 10) and that surgical LAA ligation reduces LA compliance (4, 5). Severely reduced LA compliance, especially after multiple AF ablations, can lead to stiff LA syndrome that is associated with secondary pulmonary hypertension and severe dyspnea (11–15). Increased LA stiffness has also been associated with AF recurrence after ablation (16).

The pathophysiological role of the LAA and the effect of percutaneous left atrial appendage occlusion (LAAO) upon LA mechanics are incompletely understood. In this study, we used LA PV analysis to measure changes in LA stiffness due to endocardial LAAO and to identify predictive characteristics. We devised a novel hybrid approach using near-simultaneous three-dimensional (3-D) LA volume measurements by transesophageal echocardiography (TEE) and direct invasive measurements of LA pressures.

## METHODS

### Study Population

This was a prospective study involving 27 consecutive patients evaluated at the time of percutaneous LAAO with the Watchman device (Boston Scientific, Marlborough, MA) between January 2019 and August 2019 at Yale-New Haven Hospital. Twenty-five patients were included in the final analysis after two patients were excluded. One patient with severe mitral regurgitation on intraprocedural TEE was excluded as that would confound the interpretation of the LA pressure-volume findings. One patient with intraprocedural conversion from sinus rhythm to AF was excluded to avoid confounding effects from the rhythm change. Given the invasive nature of data acquisition, no healthy volunteers were included in this study. Clinical demographics and parameters were extracted from the electronic medical record. The study was approved by the Yale University Institutional Review Board. Written, informed consent was obtained from all patients.

### Study Design

**Figure 1.**
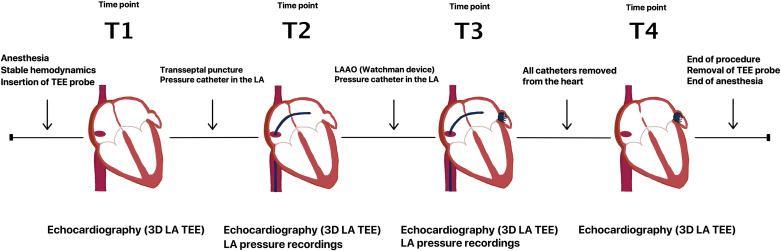
Schematic showing four intraprocedural time points during left atrial appendage occlusion (LAAO) for which pressure and/or volume data were acquired. Time points are designated as T1 (baseline), T2 (before LAAO), T3 (after LAAO), and T4 (final). LA, left atrium; TEE, transesophageal echocardiography.

We collected echocardiographic and hemodynamic data at multiple intraprocedural time points before and after Watchman device implantation for LAAO. Patients were intubated on mechanical ventilation under general anesthesia at the time of all measurements. LAAO procedures were performed without alteration of LAA size by volume loading. Standard preprocedural imaging including assessment of LAA morphology was performed by two-dimensional (2-D) TEE. LA volumes were obtained by 3-D TEE at four procedural time points (Fig. 1): baseline before transseptal puncture (*time point T1*), after access sheath and pigtail catheter placed in LA (*T2*), after Watchman device release while LA sheath still present (*T3*), and after final LA sheath removal (*T4*). Direct LA pressure measurements were obtained near simultaneously with TEE imaging at *time points T2* and *T3*. Data from *time points T2* and *T3* were obtained without any significant hemodynamic changes in between.

### Two-Dimensional TEE Data Acquisition and Analysis

We performed periprocedural TEE imaging during Watchman device placement using a Siemens ACUSON SC2000 PRIME ultrasound system with Z6Ms transducer (Siemens Medical Solutions, Issaquah, WA). Comprehensive 2-D TEE imaging was performed to obtain baseline ventricular and valvular assessments, as well as LAA size measurements (17). LAA width and depth (latter measured obliquely from landing zone midpoint to apex) at ventricular end systole were obtained at 0°, 45°, 90°, and 135° orientations. LAA size parameters were indexed by body surface area.

### Three-Dimensional TEE Data Acquisition and Analysis

**Figure 2.**
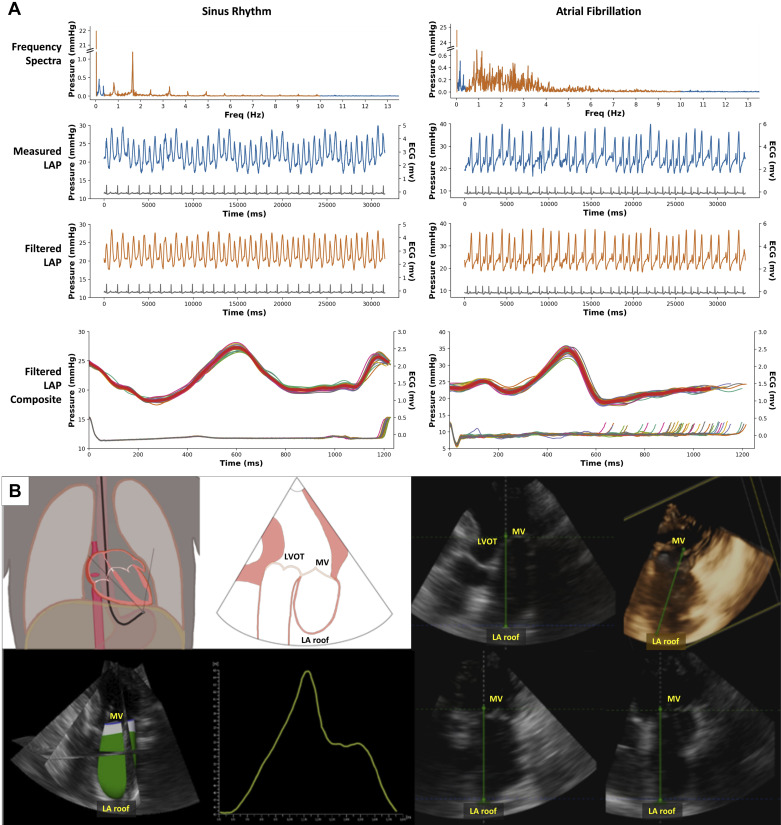
Examples of acquired pressure and volume data. *A*: left atrial pressure (LAP) acquisitions and analyses for patients in sinus rhythm (*left*) and atrial fibrillation (*right*), showing frequency spectra with filtered data in orange (*top*), measured and filtered LAP waveforms (*middle*), and filtered LAP composite waveforms for typical cardiac cycles (*bottom*). Note elimination of noise and respiratory artifact from LAP waveforms after filtering and ability to superimpose LAP waveforms with variable cycle lengths in atrial fibrillation. *B*: three-dimensional (3-D) echocardiography of LA from deep transgastric transesophageal view. *B*, *left*: schematics, LA contour, and LA volume over cardiac cycle. *B*, *right*: multiplanar views and 3-D rendering. LA, left atrium; LVOT, left ventricular outflow tract; MV, mitral valve.

LA imaging was obtained using real-time 3-D acquisitions (without multibeat gating) from a deep transgastric view allowing visualization of the entire LA (Fig. 2). Depth and spatial resolution were adjusted to achieve high volume rates (median for analyzed acquisitions was 20 volumes/s; IQR: 17–22 volumes/s). Two acquisitions were obtained at each time point. Two to three cardiac cycles were recorded for each acquisition (the latter being done with patients having irregular heart rhythms). All 3-D imaging was performed by a single operator (B.A.L.) with expertise in that technique.

Three-dimensional LA volumes were measured off-line in semiautomated fashion using TOMTEC 4 D LVA 3.0 software (TOMTEC Imaging Systems, Munich, Germany). Image quality was subjectively graded based upon ease of visual endocardial border detection. Manual corrections were applied as needed to improve contour tracking of the LA wall throughout the cardiac cycle. Acquisitions with incomplete 3-D LA visualization due to inadvertent truncation were discarded. LA volume curves were phase-averaged over multiple beats at each procedural time point (*T1* through *T4*). From these phase-averaged curves, we identified LA maximum volume (before mitral valve opening, V_max_), LA minimum volume (before mitral valve closure, V_min_), LA total emptying volume (TEV), and LA total emptying fraction (TEF) for each time point. Comparisons were made between *time points T1* and *T2* (effect of transseptal puncture with sheath present), *T2* and *T3* (effect of LAAO), *T3* and *T4* (effect of sheath removal after LAAO), and *T1* and *T4* (combined effects of LAAO and interatrial shunt creation).

### LA Pressure Data Acquisition and Analysis

We measured LA pressure after transseptal puncture at *time points T2* and *T3*. Recordings were obtained near simultaneously with TEE imaging using a fluid-filled 6-Fr Impulse pigtail catheter (Boston Scientific, Marlborough, MA) positioned in the LA through a Watchman access sheath with 14-Fr outer diameter (Boston Scientific). The catheter was connected to a leveled Transpac IV pressure transducer (ICU Medical, San Clemente, CA) and GE Mac-Lab hemodynamic recording system (GE Medical Systems, Milwaukee, WI) with a sampling rate of 977 Hz.

Pressure waveforms were exported for off-line analysis using custom scripts written in the Python 3 programming language (Python Software Foundation, https://www.python.org) using the Jupyter Notebook environment (Project Jupyter, https://jupyter.org) and SciPy library (NumFOCUS, https://scipy.org). Pressure waveforms at each time point were analyzed for 10–50 cardiac cycles. We applied the fast-Fourier transform (FFT) to each pressure waveform and used a rectangular band-pass filter between 0.4 and 10 Hz to remove respiratory variation and nonphysiological high-frequency artifacts. The mean LA pressure (nonoscillating signal magnitude at 0 Hz) was also retained. The inverse FFT was applied to recover filtered pressure waveforms. These waveforms were time-averaged with respect to the preceding R-wave. This procedure was applied for both regular and irregular rhythms (Fig. 2). From the averaged pressure waveforms, we identified mean LA pressure (LAP_mean_), maximum LA pressure (LAP_max_), minimum LA pressure (LAP_min_), v-wave peak pressure, time to v-wave peak (relative to preceding R-wave peak on ECG), and linear dP/d*T* (change in pressure with change in time) of the v-wave ascending limb (LA filling).

### Pressure-Volume Loop Analysis

**Figure 3.**
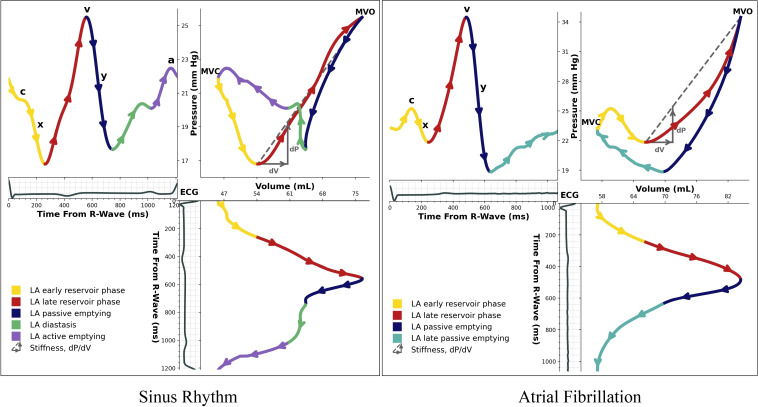
Left atrial (LA) pressure-volume loop analysis for patients in sinus rhythm (*left*) and atrial fibrillation (*right*). Arrows show progression through cardiac cycle, and colors indicate phases: yellow, filling during early reservoir phase; red, filling during late reservoir phase; blue, passive emptying; green, diastasis; purple, active emptying in sinus rhythm; turquoise, late passive emptying in atrial fibrillation. MVC, mitral valve closure; MVO, mitral valve opening.

Pressure and volume curves were interpolated to equivalent temporal resolutions that also matched the average cardiac cycle length during TEE volume acquisitions (median 999 points per PV loop; IQR: 937–1,078 points). As echocardiographic acquisitions have an inherent temporal ambiguity (each volume must be assigned to a single instant in time, but beam lines are actually acquired continuously over the entire sampling interval), we determined an appropriate temporal correction for each pressure-volume pair by matching the v-wave peak pressure to the LA maximum volume (both corresponding to the time just before mitral valve opening). This allowed the mapping of a PV loop relationship for each pair. We measured LA stiffness (in mmHg/mL) by determining the linear slope of dP/dV derived from the endpoints of the clockwise ascending limb of LA filling. This approach was chosen because the pressure-volume curves did not exhibit a universally consistent exponential or curvilinear shape in that region (Fig. 3).

### Cohort Analyses

We performed cohort analyses to evaluate factors that might be expected to influence the magnitude of decreased compliance after Watchman device placement. The first analysis involved dividing the study population into groups designated as having either paroxysmal (for patients presenting in sinus rhythm) or persistent AF (for patients presenting in AF). All parameters were compared for significant differences between these groups. The second analysis involved dividing the study population into halves with respect to baseline LA pressure (based upon preimplantation mean LA pressure at T2) and comparing stiffness parameters between the groups designated as having low or high LA pressure. The third and fourth analyses involved dividing the study population into halves with respect to either baseline LA size (based upon preimplantation-indexed maximum LA volume at T1) or baseline LAA size (by indexed maximum depth) and comparing stiffness parameters between the groups designated as having small or large sizes.

### Intraobserver and Interobserver Reproducibility

We investigated the reproducibility of evaluating LA volumes by 3-D TEE by assessing reliability for the primary observer (A.B.) and between primary and secondary (X.A.O.) observers. Both observers are experienced echocardiographers. The primary observer evaluated TEE images from all patients and time points. These measurements were used in the overall study analysis. Two months after the initial measurements, the primary and secondary observers reevaluated baseline time-point images in a blinded fashion for 10 randomly selected patients each.

Given the complicated form of the volume curves, comparisons were facilitated by using four discrete parameters to describe each volume curve during LA filling. The first two parameters were minimum and maximum LA volumes. The last two parameters were the initial value *A* and growth constant *K* obtained after fitting each LA-filling volume curve to an exponential function with form V = *A*e*^Kt^*, where V is volume, *A* is initial value, e is Euler number, *K* is growth constant, and *t* is time (Supplemental Fig. S1; all Supplemental material is available at https://doi.org/10.6084/m9.figshare.20469459).

### Statistical Analysis

Analyses were performed using custom scripts written in the Python 3 programming language (Python Software Foundation, https://www.python.org) using the Jupyter Notebook environment (Project Jupyter, https://jupyter.org) and SciPy library (NumFOCUS, https://scipy.org). Reproducibility testing was performed using statistical packages (epiR and psych) for the R programming language.

Nonparametric analyses were used as no variables showed normal distribution (based upon Shapiro–Wilk normality test). All data are reported as median values with interquartile ranges. We used two-tailed Wilcoxon signed-rank test for paired and Mann–Whitney–Wilcoxon test for unpaired comparisons. Differences were considered significant for *P* < 0.05 (presented along with 95% confidence intervals estimated by bootstrapping). For paired sample LA volume comparisons with multiple time-point pairings, Bonferroni correction was used, and differences were considered significant for *P* < 0.0125 (listed along with 98.75% confidence intervals estimated by bootstrapping). Gardner–Altman and Cumming plots were used for data presentation (18). Spearman’s rank correlation coefficient *R*_s_ was used to assess correlations between variables, and these were considered significant for *P* < 0.05.

Intraobserver reliability was assessed by calculating the intraclass correlation coefficient (with 95% confidence interval) for two-way mixed effects for absolute agreement with a single observer [ICC (3,1)]. Interobserver reliability was assessed by calculating the intraclass correlation coefficient (with 95% confidence interval) for two-way random effects for absolute agreement between two observers [ICC (2,1)] (19).

## RESULTS

### Patient Characteristics

Demographic and clinical characteristics are shown in Table 1. Three-dimensional LA image quality was rated as low (but acceptable) for 3 patients (12%), moderate for 17 patients (68%), and high for 5 patients (20%). After LAAO, four patients (16%) had a small residual leak around the Watchman device (each measured as 1 mm in width by color flow).

**Table 1.** Patient demographics

Variables	
Patients undergoing LAAO, *n* (%)	25
Age, yr	80 [71–83]
Males, *n* (%)	17 (68)
Height, cm	173 [160–180]
Weight, kg	92 [73–100]
BSA, m^2^	2.1 [1.8–2.2]
Prior atrial fibrillation ablation, *n* (%)	1 (4)
Rhythm, *n* (%)	
Paroxysmal atrial fibrillation	12 (48)
Persistent atrial fibrillation	13 (52)
Hypertension, *n* (%)	22 (88)
Diabetes mellitus, *n* (%)	12 (48)
Hyperlipidemia, *n* (%)	19 (76)
CAD, *n* (%)	13 (52)
CHF, *n* (%)	5 (20)
COPD, *n* (%)	5 (20)
LV function preserved (LVEF ≥50%), *n* (%)	22 (88)
RV function normal, *n* (%)	21 (84)
Mitral regurgitation, *n* (%)	
Moderate	3 (12)
Less than moderate	22 (88)
3-D LA image quality, *n* (%)	
Low	3 (12)
Moderate	17 (68)
High	5 (20)
Post-LAAO residual leak around Watchman device, *n* (%)	4 (16)

Values are medians [interquartile ranges] or number (*n*) of subjects (percentages). BSA, body surface area; CAD, coronary artery disease; CHF, congestive heart failure; COPD, chronic obstructive pulmonary disease; LA, left atrial; LAAO, left atrial appendage occlusion; LV, left ventricular; RV, right ventricular.

### LA Pressure

Results for the overall study population are shown in Table 2 (and Supplemental Fig. S2). LA filling dP/d*T* significantly increased after device placement when compared with before (*P* ≪ 0.001). Time to v-wave peak significantly decreased (*P* ≪ 0.001). There were no significant changes in cardiac cycle length, v-wave peak pressure, or LA pressures.

**Table 2.** LAP and volume measurements before and after LAAO

	Time Points			
Variables	Baseline (T1)	Before LAAO (T2)	After LAAO (T3)	Final (T4)
Volumes				
LA V_max_ indexed, mL/m^2^	44.6* [32.1, 55.1]	47.2 [35.1, 57.0]	49.8 [33.5, 58.2]	48.3* [34.9, 63.1]
LA V_min_ indexed, mL/m^2^	31.3 [19.9, 38.6]	35.4 [22.9, 40.6]	35.3 [23.9, 43.9]	30.9 [20.9, 43.2]
LA TEV indexed, mL/m^2^	13.2* [11.5, 18.0]	13.7 [12.5, 16.7]	12.8† [10.5, 15.0]	17.4*† [14.4, 19.8]
LA TEF	0.31 [0.29, 0.37]	0.30 [0.25, 0.38]	0.27† [0.24, 0.32]	0.34† [0.29, 0.42]
Pressures				
RR length, ms		999 [937, 1,078]	955 [873, 1,109]	
Time to V_peak_, ms		540‡ [512, 569]	503‡ [486, 522]	
Time to V_peak_ adjusted for RR, %		54.1‡ [50.1, 59.4]	51.2‡ [46.4, 56.2]	
V_peak_, mmHg		28.6 [23.9, 35.6]	29.5 [26.3, 35.5]	
LA filling dP/d*T*, mmHg/s		29.0‡ [20.7, 47.5]	39.0‡ [26.4, 47.9]	
Mean LAP, mmHg		23.4 [21.1, 26.5]	22.4 [20.9, 28.9]	
Maximum LAP, mmHg		28.6 [26.4, 35.6]	29.5 [27.5, 35.5]	
Minimum LAP, mmHg		18.4 [16.6, 20.8]	18.8 [16.2, 20.6]	

Values are medians [interquartile ranges]. LA, left atrial; LAP, LA pressure; LAAO, LA appendage occlusion; RR length, cardiac cycle length; T1, baseline time point before transseptal puncture; T2, time point with only LA sheath present; T3, time point with LA sheath present after device release; T4, final time point after LA sheath removal; TEF, total emptying fraction; TEV, total emptying volume; V_max_, maximum volume; V_min_, minimum volume; V_peak_, v-wave peak. Significance symbols for paired sample comparisons by Wilcoxon signed-rank test for volumes with *P* < 0.0125 (significance threshold determined by Bonferroni correction) and for pressures with *P* < 0.05 for following time points: *T1–T4; †T3–T4; ‡T2–T3.

Cohort analysis results by rhythm showed that, at *time point T2* (before device placement), the paroxysmal AF group showed significantly lower mean LA pressure (*P* = 0.02), lower maximum LA pressure (*P* < 0.01), and lower v-wave peak pressure (*P* < 0.01) than the persistent AF group (Supplemental Fig. S3).

### LA Volume and Function

Results are shown in Table 2 (and Supplemental Fig. S4). Comparison between *time points T1* (baseline) and *T4* (after final LA sheath removal) showed increased indexed maximum LA volume (*P* < 0.01) and increased indexed LA total emptying volume (*P* < 0.01). Comparison between *time points T3* (after LAAO, but before LA sheath removal) and *T4* (after final LA sheath removal) showed increased indexed LA total emptying volume (*P* < 0.001) and increased LA total emptying fraction (*P* < 0.001). Comparison between *time points T2* (before LAAO) and *T3* (after LAAO), both with LA sheath present, showed no significant changes.

Cohort analysis by rhythm showed that at *time point T1* (baseline), the paroxysmal AF group showed significantly lower indexed maximum LA volume (*P* < 0.01), lower indexed minimum LA volume (*P* < 0.001), and higher LA total emptying fraction (*P* < 0.001) than the persistent AF group (Supplemental Fig. S5).

### Baseline LA Appendage Size

Median values for baseline LA appendage size included indexed maximum width of 1.02 cm/m^2^ [IQR, 0.95–1.13 cm^2^/m^2^] and indexed maximum depth of 1.38 cm/m^2^ [IQR, 1.25–1.55 cm^2^/m^2^]. There were no significant differences in indexed LA appendage size measurements when comparing the paroxysmal and persistent AF groups. Results are shown in Supplemental Table S1.

### LA Stiffness

**Figure 4.**
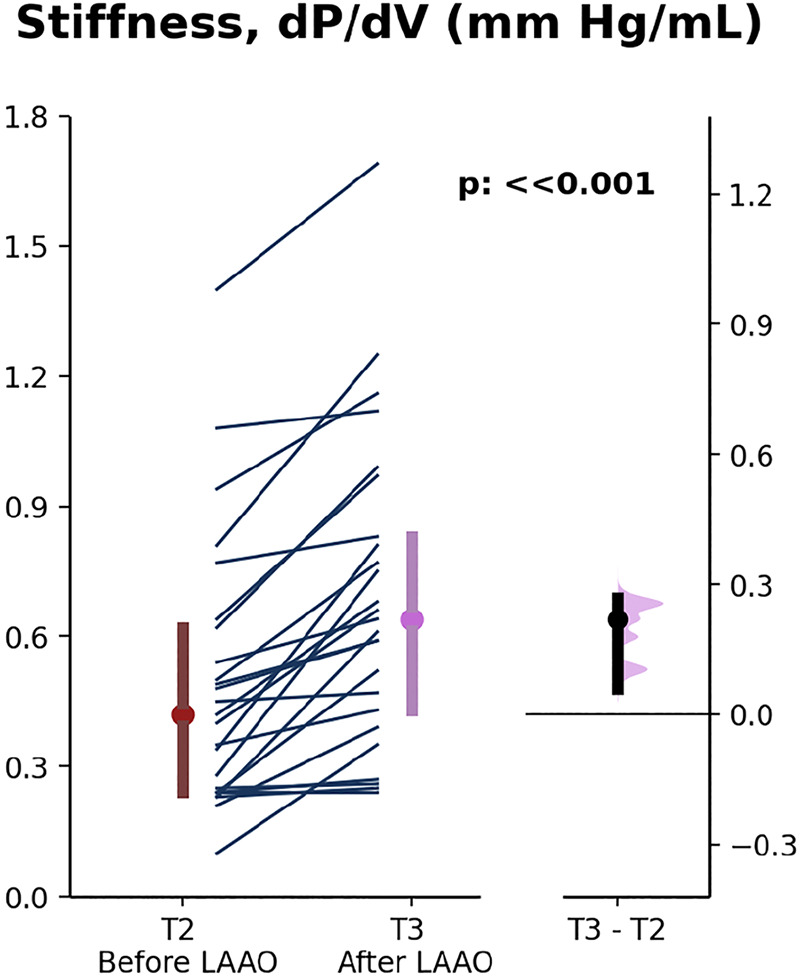
Left atrial (LA) stiffness changes before and after left atrial appendage occlusion (LAAO). Gardner–Altman plots with all data points represented as slope graphs with median values and interquartile ranges. Median differences are shown on right along with 95% confidence intervals. T2, time point with only LA sheath present; T3, time point with LA sheath present after device release.

**Figure 5.**
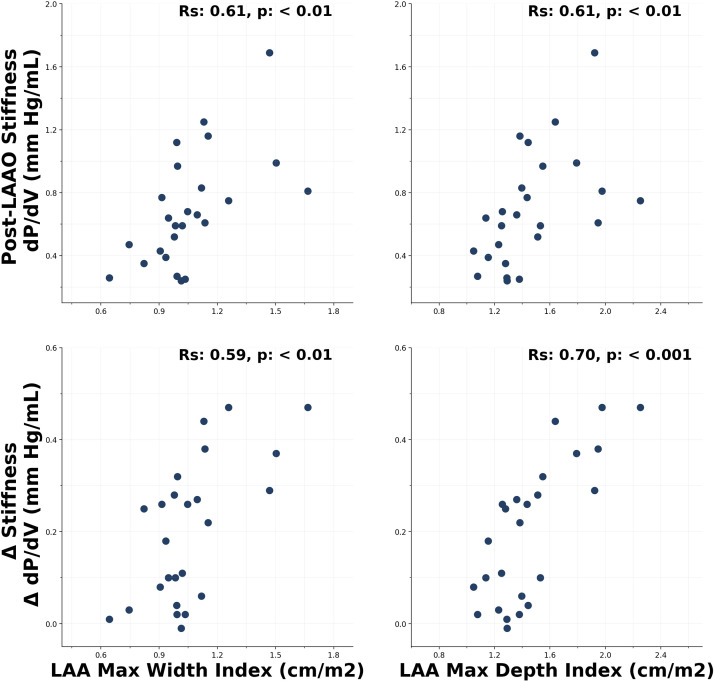
Baseline left atrial appendage (LAA) size correlation with left atrial stiffness and stiffness change after left atrial appendage occlusion (LAAO). Scatter plots are shown with Spearman’s rank (*R*_s_) correlation coefficients. *P* < 0.05 considered significant.

LA stiffness significantly increased after device placement when compared with before (median, 0.41–0.64 mmHg/mL; *P* ≪ 0.001; Fig. 4 and Table 3). Correlation with baseline LAA size was strongest with indexed LAA maximum depth for post-LAAO stiffness change (*R*_s_ = 0.70; *P* < 0.001; Fig. 5). LA stiffness before and after LAAO correlated with maximum LA pressure, but no correlations were seen with mean LA pressure or baseline LA size (Supplemental Table S2).

**Table 3.** LA stiffness measurements before and after LAAO

	Stiffness, dP/dV, mmHg/mL			ΔStiffness,
Variables	Before LAAO (T2)	After LAAO (T3)	*P* (W)	ΔdP/dV, mmHg/mL
All patients				
All	0.41 [0.24, 0.62]	0.64 [0.43, 0.83]	**<0.001**	0.22 [0.06, 0.29]
Rhythm				
Paroxysmal	0.40 [0.25, 0.49]	0.59 [0.47, 0.66]	**<0.001**	0.11 [0.08, 0.28]
Persistent	0.46 [0.24, 0.78]	0.79 [0.33, 1.02]	**<0.01**	0.26 [0.05, 0.30]
*P* (MWW)	0.27	0.18		0.44
LAP_mean_, mmHg				
Low [13.8–23.4]	0.28 [0.24, 0.45]	0.47 [0.27, 0.64]	**<0.01**	0.10 [0.02, 0.37]
High [23.4–37.5]	0.50 [0.40, 0.81]	0.77 [0.59, 1.12]	**<0.001**	0.26 [0.11, 0.28]
*P* (MWW)	**0.03**	**0.02**		0.16
LA V_max_ indexed, mL/m^2^				
Small [24.6–44.6]	0.45 [0.25, 0.54]	0.59 [0.47, 0.75]	**<0.001**	0.18 [0.10, 0.28]
Large [44.6–88.5]	0.42 [0.24, 0.77]	0.77 [0.43, 0.99]	**<0.01**	0.26 [0.06, 0.37]
*P* (MWW)	0.45	0.24		0.39
LAAD indexed, cm/m^2^				
Small [1.05–1.38]	0.35 [0.24, 0.48]	0.47 [0.35, 0.64]	**<0.001**	0.10 [0.03, 0.25]
Large [1.38–2.25]	0.49 [0.28, 0.77]	0.81 [0.61, 0.99]	**<0.001**	0.29 [0.22, 0.38]
*P* (MWW)	**0.02**	**<0.001**		**<0.01**

Values are medians [interquartile ranges] except for cohort groupings listed with lower and upper limits. AF, atrial fibrillation; dP/dV, change of pressure with change of volume; LA, left atrial; LAP, LA pressure; LAAD, LA appendage depth; LAAO, LA appendage occlusion; MWW, Mann–Whitney–Wilcoxon test; T2, time point with only LA sheath present; T3, time point with LA sheath present after device release; V_max_, maximum volume; W, Wilcoxon signed-rank test. *P* < 0.05, significant values shown in boldface.

Results for the cohort analyses related to rhythm, baseline LA pressure, baseline LA size, and baseline LAA size are shown in Table 3. For all cohorts, LA stiffness significantly increased after device placement when compared with before (*P* < 0.01 in all cases). For the large LAA size group, the magnitude of LA stiffness increase after LAAO was significantly higher than the small LAA size group (*P* < 0.01). No significant differences were seen for stiffness parameters when patients were categorized into groups based upon rhythm or baseline LA size.

### Intraobserver and Interobserver Reproducibility

Intraclass correlation coefficients for maximum and minimum LA volumes as well as initial value *A* were good to excellent for both intraobserver and interobserver reliability (ranging from 0.85 to 0.98). Intraclass correlation coefficients for growth constant *K* were moderate for both intraobserver and interobserver reliability (0.71 and 0.57, respectively; Supplemental Figs. S1 and S6).

## DISCUSSION

To study LA mechanics, we devised a novel hybrid approach to perform PV analysis to measure LA stiffness before and immediately after endocardial LAAO. LA stiffness increased significantly after device placement. Baseline LAA size correlated with final stiffness parameter values and the magnitudes of the increases. Median LA stiffness increased from 0.41 to 0.64 mmHg/mL pre- and post-LAAO, respectively. Of note, Reddy et al. (8) reported results equivalent to median LA stiffness of 0.18 mmHg/mL in controls, but 0.45 and 0.59 mmHg/mL in patients with heart failure combined with either paroxysmal or permanent AF, respectively. Their data highlight the correlation between increased LA stiffness and cardiac disease severity. In our study population, chronic pathophysiological changes led to abnormal LA body stiffness that became dominant after the loss of LAA compliance due to LAAO. Pre-LAAO stiffness parameters are due to combined LA body and LAA properties, whereas post-LAAO stiffness parameters are due to the LA body alone. Baseline LAA size correlation with post-LAAO stiffness parameters supports the concept that the LAA is remodeled in response to chronically increased LA body stiffness. To our knowledge, this is the first study to document a relationship between LAA size and LA body stiffness, as well as changes in overall mechanics after endocardial LAAO. LAA size correlates with LA body stiffness better than LA size does and may potentially be a superior clinical predictor. Future studies will be needed to define normal and abnormal ranges for LAA size by different methodologies (including TEE, CT, or MRI) and to establish correlations with clinical outcomes.

It has been shown that surgical LAA ligation increases the incidence of postoperative atrial fibrillation, possibly because of decreased ability to tolerate volume shifts (20). This suggests that LAA distensibility may allow it to serve as a physiological “pop-off valve” to mitigate abnormal pressure changes with volume overload. Acute and chronic neurohormonal response patterns after percutaneous LAAO are different for epicardial (analogous to surgical closure) and endocardial approaches (7). It is not yet clear how reduced LA compliance after LAAO might affect responses to chronic volume challenges and subsequent LA remodeling.

Severely reduced LA compliance can lead to stiff LA syndrome with important clinical consequences (11–15). When associated with severe dyspnea, atrial septostomy has even been advocated as a potential therapy (15). LA stiffness increases after AF ablation (21) and patients with multiple ablations are at increased risk for stiff LA syndrome (13). Increased LA stiffness at the time of AF ablation is associated with future AF recurrence (16). Notably, LAA size increases after AF ablation and also correlates with future AF recurrence (22, 23). Given the 55% relative increase in median LA stiffness found in our study, LAAO may also be a risk factor for stiff LA syndrome in vulnerable patients. AF ablation and LAAO procedures have well-known benefits such as rhythm control and thromboembolic risk reduction strategies. As there is considerable overlap between patient populations undergoing these two procedures, it will be important to expand our understanding of associated short- and long-term hemodynamic changes.

Intraprocedural changes in LA pressure and volume dynamics were noted in our study. LA filling dP/d*T* increased and time to v-wave peak decreased, whereas mean and peak LA pressures did not change. This suggests that pressure dynamics are more sensitive than absolute pressures to global changes in LA mechanics. LA size (indexed LA V_max_) increased between *time points T1* and *T4*. There was also an increase in LA emptying volume between *T1* and *T4* (but not between *T2* and *T3*) that was most likely due to iatrogenic interatrial shunt creation, as there was no significant change seen until the LA sheath was removed.

Changes in LA size and function after LAAO have been discussed in the literature. LA size appears to increase after endocardial LAAO and decrease after epicardial LAAO (24–28). Although both procedures involve displacement of LAA volumes and interatrial shunt creation, the discrepancy may be explained by the fact that endocardial LAAO preserves the LAA orifice, whereas epicardial LAAO collapses it. The resulting geometric alteration likely leads to reduced effective LA body surface area, radius, and volume. Volumetric and strain imaging-based indices have been used to evaluate LA function, but the results have not been consistent. Murtaza et al. (27) found that LA volumetric indices were increased but LA strain indices did not change after endocardial LAAO, whereas Dar et al. (28) found increased LA strain rates after epicardial LAAO. Creation of an iatrogenic interatrial shunt, however, after either endocardial or epicardial LAAO should increase LA emptying volumes (as seen in our study). This would confound attempts to compare LA function before and after LAAO by either volumetric or strain methods, while PV analysis of LA compliance (before transseptal sheath removal) would not be affected.

Our study introduces technical advances not previously described in the literature. Single-beat wide-sector 3-D LA volumes obtained from a deep transgastric TEE approach allowed visualization of the entire LA at high volume rates intraprocedurally without spatial truncation or geometric assumptions (29). Off-line FFT processing of pressure tracings allowed the elimination of noise and respiratory motion artifacts. Time averaging (rather than phase averaging) of waveforms allowed reconstruction of “typical” pressure waveforms for patients in atrial fibrillation. This involved superimposing truncated waveforms with respect to time from the preceding R-wave.

Although some variation in echocardiographic image quality was noted and sometimes required multiple acquisition attempts to find the optimal acoustic window, adequate visualization for endocardial border detection was ultimately feasible for all patients in this study. LA volume curves were measured without including the LA appendage volume since the latter is not easily obtained by echocardiography. As the LA appendage is more compliant than the LA body, not including dynamic LA appendage volumes may actually slightly overestimate baseline LA stiffness and underestimate the amount of LA stiffness increase after LAAO.

Limitations of this study include a relatively small sample size. Despite this, we saw very consistent trends across several variables of interest. This study was designed to assess intraprocedural changes and did not include outpatient follow-up. Future studies are needed to examine long-term clinical implications.

In conclusion, using a novel hybrid PV analysis combining direct LA pressure measurements with 3-D TEE LA volumes, we found that overall LA stiffness increases after endocardial LAAO. Baseline LA appendage size correlates with the magnitude of increase and LA body stiffness. These findings document alteration of LA mechanics after endocardial LAAO and suggest that the LA appendage modulates overall LA compliance.

## SUPPLEMENTAL DATA

Supplemental Figs. S1–S6 and Supplemental Tables S1–S2: https://doi.org/10.6084/m9.figshare.20469459.

## GRANTS

Dr. Bregasi was supported by the Julio F. Tubau Fund.

## DISCLOSURES

Dr. J. V. Freeman serves on the advisory board for Boston Scientific. None of the other authors has any conflicts of interest, financial or otherwise, to disclose.

## AUTHOR CONTRIBUTIONS

A.B. and B.A.L. conceived and designed research; A.B., J.V.F., J.P.C., J.G.A., and B.A.L. performed experiments; A.B., X.A.O.-L., J.H.M., A.Y.H., and B.A.L. analyzed data; A.B. and B.A.L. interpreted results of experiments; A.B. prepared figures; A.B. and B.A.L. drafted manuscript; A.B., J.V.F., J.P.C., J.G.A., X.A.O.-L., J.H.M., A.Y.H., R.V.M., A.J.S., R.L.M., L.S., and B.A.L. edited and revised manuscript; A.B., J.V.F., J.P.C., J.G.A., X.A.O.-L., J.H.M., A.Y.H., R.V.M., A.J.S., R.L.M., L.S., and B.A.L. approved final version of manuscript.
